# Variation in *E. coli* concentrations in open drains across neighborhoods in Accra, Ghana: The influence of onsite sanitation coverage and interconnectedness of urban environments

**DOI:** 10.1016/j.ijheh.2019.113433

**Published:** 2020-03

**Authors:** David M. Berendes, Laura de Mondesert, Amy E. Kirby, Habib Yakubu, Lady Adomako, James Michiel, Suraja Raj, Katharine Robb, Yuke Wang, Benjamin Doe, Joseph Ampofo, Christine L. Moe

**Affiliations:** a Waterborne Disease Prevention Branch, Division of Foodborne, Waterborne, and Environmental Diseases, Centers for Disease Control and Prevention, Atlanta, GA, USA; b Center for Global Safe Water, Sanitation, and Hygiene, Rollins School of Public Health, Emory University, Atlanta, GA, USA; c Council for Scientific and Industrial Research, Water Research Institute, Accra, Ghana; d Training, Research, and Networking for Development (TREND) Group, Accra, Ghana

**Keywords:** Sanitation, Urban environments, WASH, Low- and middle-income countries, Fecal contamination

## Abstract

Alongside efforts to improve safe management of feces along the entire sanitation chain, including after the toilet, global sanitation efforts are focusing on universal access ‘basic’ services: onsite facilities that safely contain excreta away from human contact. Although fecal sludge management is improving in urban areas, open drains remain a common fate for feces in these often densely-populated neighborhoods in low-income countries. To-date, it is unclear to what extent complete coverage of onsite sanitation reduces fecal contamination in the urban environment and how fecal contamination varies within urban drains across neighborhoods by sanitation status within a city. We assessed how neighborhood levels of environmental fecal contamination (via spatially-representative sampling of open drains for *E. coli*) varied across four neighborhoods with varying income, type and coverage of household sanitation facilities, and population density in Accra, Ghana. Neighborhoods with very high sanitation coverage (≥89%) still had high (>4 log_10_ CFU/100 mL) *E. coli* concentrations in drains. Between-neighborhood variation in *E. coli* levels among the high coverage neighborhoods was significant: drain concentrations in neighborhoods with 93% and 89% coverage (4.7 (95% CI: 4.5, 4.9) & 4.9 (95% CI: 4.5, 5.3) log_10_ CFU/100 mL, respectively) were higher than in the neighborhood with 97% coverage (4.1 log_10_ CFU/100 mL, 95% CI: 3.8, 4.4 log_10_ CFU/100 mL). Compared with the highest coverage neighborhood, the neighborhood with lowest coverage (48%) also had higher *E. coli* concentrations (5.6 log_10_ CFU/100 mL, 95% CI: 5.3, 5.9 log_10_ CFU/100 mL). Although fecal contamination in open drains appeared lower in neighborhoods with higher onsite sanitation coverage (and vice versa), other factors (e.g. fecal sludge management, animals, population density) may affect drain concentrations. These results underscore that neighborhood-level onsite sanitation improvements alone may not sufficiently reduce fecal hazards to public health from open drains. These findings supporting the need for integrated, city-level fecal sludge management alongside multifaceted interventions to reduce fecal contamination levels and human exposure.

## 1 Introduction

Open drains remain ubiquitous vessels for rainwater and graywater, but they also serve as conduits for feces from onsite, decentralized sanitation systems (primarily those that are unsafely managed, in low-income, urban environments). Exposure to open drain water may be an important risk factor for enteric infections in these environments (Gretsch et al., 2015; Katukiza et al., 2013; Labite et al., 2010).

Improvements in the coverage and quality of decentralized sanitation systems that ensure feces are contained away from human contact (onsite/at the household), safely emptied from household containment infrastructure and treated, or otherwise safely-managed, are hypothesized to reduce environmental fecal contamination (Wagner and Lanoix, 1958) and therefore are a focus of global policy and monitoring systems. Globally, the Sustainable Development Goals (SDGs (United Nations, 2015)) for sanitation focus on the need for “safely-managed sanitation”: safe management of feces along the entire sanitation chain, including abandoning open drainage as a discharge and conveyance option (World Health Organization, 2018), but also emphasize universal “basic” coverage (i.e. of onsite systems that contain excreta away from human contact). High coverage of drainage and sewerage infrastructure has been shown, in most cases, to reduce pediatric diarrheal risks in some urban environments (Moraes et al., 2003; Norman et al., 2010; Wolf et al., 2018), though not all cases, such as those with poor levels of service (Roushdy et al., 2012). Safe management remains challenging to measure—primarily because of limitations in methods for efficiently estimating how much waste from households is adequately treated in—and therefore is difficult to improve. Most estimates of safe-management in low-income countries are incomplete: the highest level of the sanitation ladder in LMICs currently combines “safely-managed” and “basic” into “at least basic”, which operationally encompasses at least “improved” (per previous Millennium Development Goal (MDG) definitions) private facilities (UNICEF and WHO, 2017). Globally, at least 66% of urban populations use onsite sanitation that generally requires emptying (D. M. Berendes et al., 2017). If not properly sited or if otherwise “improved” or “basic” sanitation facilities are unsafely emptied, these facilities may continue to contaminate open drains. Therefore, measurement of downstream emptying and treatment is imperative. Urban drains may also receive fecal contamination from open defecation or other sources, including domestic and feral animals. Given the current challenges in quantifying safe management (that restrict measurement to quantification of household-level sanitation characteristics) and the focus on achieving universal basic coverage (UNICEF and WHO, 2017), there is a need to understand how environmental fecal contamination varies in urban settings, including drains, and whether improvements to household sanitation coverage alone can reduce environmental fecal contamination (e.g. in drains).

The goal of this study was to assess neighborhood-level fecal contamination in open drains and its variation between neighborhoods with differing coverage and type of household-level sanitation using household surveys and spatially-representative sampling of open drains in Accra, Ghana. Results from these densely-populated urban environments can improve understanding of fecal contamination levels in urban settings and how fecal contamination at the neighborhood-level may vary with coverage of household-level sanitation in other contexts. Additionally, results can begin to quantify the extent that sanitation coverage measured at the household alone may reduce environmental contamination.

## 2 Materials and methods

### 2.1 Study area

**Fig. 1:**
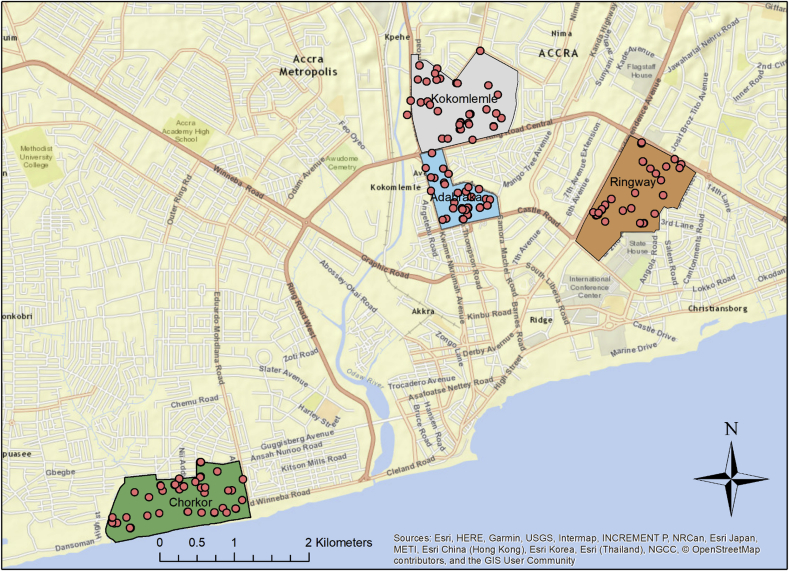
Locations of study neighborhoods (polygons) and drain sampling locations (dots), Accra, Ghana. ©OpenStreetMap contributors. Data available under the Open Database License, for more information: https://www.openstreetmap.org/copyright.

This cross-sectional study was conducted in four neighborhoods (Adabraka, Chorkor, Kokomlemle, and Ringway Estates, Fig. 1) of Accra, Ghana, in collaboration with the Water Research Institute of the Center for Scientific and Industrial Research Institute, Ghana (WRI) and the Training, Research, and Networking for Development (TREND) Group, from March–July 2016 as part of a SaniPath Exposure Assessment (Emory University, 2014; Robb et al., 2017). The SaniPath Exposure Assessment Tool examines environmental pathways of exposure to fecal contamination in urban neighborhoods using both behavioral and microbiological data (Emory University, 2014; Robb et al., 2017). Neighborhoods were purposively selected for variation in levels of sanitation coverage, population density, and socioeconomic status based on the 2010 Ghana Population and Housing census from all neighborhoods in that census (CHF International et al., 2010; Ghana Statistical Service, 2014). Ringway Estates was a ‘non-poverty’ income area (>$10/day), while Adabraka and Kokomlemle were ‘moderate poverty’ ($2–5/day), and Chorkor was ‘high poverty’ ($1–2/day) (CHF International et al., 2010). Ringway Estates had ‘low’ population density (5000–10,000 people/km^2^, total population: 2825 (Ghana Statistical Service, 2014)), Adabraka had ‘high’ population density (20,000–30,000/km^2^, total population: 36,510 (Ghana Statistical Service, 2014)), and Kokomlemle and Chorkor had ‘very high’ population density (>30,000/km^2^, total populations of 35,320 and 26,855, respectively (Ghana Statistical Service, 2014)) (CHF International et al., 2010). All study protocols and documents were approved by the Emory University Institutional Review Board (IRB #00051584) and the University of Ghana IRB (IRB #00001276).

Accra receives approximately 760 mm of rainfall each year, and a year-round mean temperature of 20°–30 °C (Gretsch et al., 2015). The rainy seasons occur in March–July (the wettest months) and in August–October (Adank et al., 2011). Ghana's most recent census report (2010) indicated that the average household size within the Greater Accra Metropolitan Area was approximately 3.7 persons per household (Ghana Statistical Service, 2014).

### 2.2 Household surveys

Within each neighborhood, 150 households were targeted for survey, with the exception of Chorkor where 200 households were targeted for survey to allow for other sub-analyses of the data collected by the SaniPath Exposure Assessment Tool (Emory University, 2014). All households were selected using combined systematic and random sampling methods. Neighborhoods were geographically divided into quadrants of approximately equal area to maximize coverage of surveys and ensure spatial heterogeneity within sub-populations. All households were then enumerated along each street or alley within the quadrant. Enumerators then worked in pairs to randomly select a house or compound (structure) at the end of a street or alley of the grid, administer the survey, and then move at an interval of eight to the next house or compound for interview. Within a house or compound, the first household met by the enumerator was interviewed. If the household refused, the next household was approached, and so on, until the survey interview was successfully conducted or no more households were available. A household was defined as any living space occupied by a group of two or more people who share the same cooking pot or meal.

Enumerators administered surveys in pairs and collected GPS coordinates at each household using a CommCare mobile app (Dimagi, Cambridge, MA, USA) on a mobile phone. Surveys were administered to the person responsible for water, sanitation, and hygiene (WASH) activities, generally the female household head. If a household toilet was reported, enumerators observed the toilet and classified it according to the Joint Monitoring Programme (JMP) definitions (UNICEF and WHO, 2017). Subsequently, household sanitation was reclassified into “contained” and “uncontained” toilets. Contained toilets consisted of flush toilets or pour flush toilets to sewerage or contained onsite systems, Kumasi ventilated improved pit [KVIP] latrines, ventilated improved pit [VIP] latrines, and traditional pit latrines. Uncontained toilets consisted of buckets, pans, and those reporting no toilet and subsequently open defecating. We defined a contained toilet as one that met the infrastructure-based criteria for “improved” (MDG) or “basic” (SDG) sanitation based on its physical characteristics because, according to JMP definitions (UNICEF and WHO, 2017), it was designed to provide safe disposal and physical separation of excreta from human contact. Households with toilets were asked whether they shared the toilet with any other households, and if so, how many. Toilets that were shared by multiple households would not be classified as “improved” or “safely-managed” by JMP definitions (under the SDG criteria, they are classified as ‘limited’ sanitation (UNICEF and WHO, 2017)). *A priori,* given previous knowledge of the extent of shared sanitation facilities in Accra (Berendes et al., 2018a; Mazeau et al., 2013; Peprah et al., 2015), we anticipated that many toilets in this study would be shared and therefore we would have insufficient sample size of ‘private’ toilets in the study areas to separate them (basic sanitation) from limited/shared sanitation for analysis. Consequently (and because our focus was on toilet infrastructure), we used the “contained” vs. “uncontained” terminology. Frequency of public toilet use per week was also assessed.

### 2.3 Drain water sample collection and processing

Drain water sampling locations within each study neighborhood were selected spatially at random *a priori* using a spatial grid of each neighborhood. Study neighborhoods were divided into 10 × 10 m square grids in ArcGIS version 10.2 (Esri, Redlands, CA, USA). In each neighborhood, 30 grids were randomly selected for collection of a single drain sample, while an additional 5 grids were randomly selected for collection of two samples in a single day (1 morning, 1 afternoon) to assess temporal variation for a separate sub-study, for a total of 40 samples (160 samples total) from 35 unique sites per neighborhood (140 unique sites total). For each of the initial 30 samples per neighborhood, teams collected 100 mL of drain water at a sample collection site randomly-selected by geolocation within the grid and recorded observations of environmental conditions at the time of collection (e.g. rainfall at the time of collection, size of the drain, and other conditions). If no drains were present within a selected grid, the team sampled from the drain located nearest to the grid in any direction. All drains sampled were along roads. For the additional 5 squares that were sampled twice in a day, teams collected 100 mL of drain water at a randomly-selected sample collection site and recorded conditions as above. Subsequently, they ensured they returned to that exact site in the afternoon to collect a second replicate sample. Samples for the larger study comparing across neighborhoods were collected in the morning (8 h–12 h); the subset of samples that were repeated from the same sites were collected in the afternoon (12 h–14 h) of the same day for within-day comparison of *E. coli* concentrations. For all sampling sites, data and GPS coordinates were collected using a CommCare mobile app (Dimagi, Cambridge, MA, USA) on a mobile phone. All samples were tested for *E. coli* concentrations as an indicator of fecal contamination (Gruber et al., 2014; Tallon et al., 2005).

Collection protocols have been described previously (Emory University, 2014; Robb et al., 2017). Briefly, drain samples were collected using a sterile bailer or Sludge Nabber (Nasco, Fort Atkinson, WI, USA) to scoop drain water into sterile 100 mL Whirl-Pack bags (Nasco, Fort Atkinson, WI, USA), which were sealed and transported in coolers with ice packs to the WRI laboratory within 8 h of collection. Samples were stored at 4 °C until analysis. Drain samples were analyzed by membrane filtration for *E. coli* according to United States Environmental Protection Agency (USEPA) Method 1604 (USEPA, 2002) with mColiBlue (Millipore Sigma, Darnstadt, Germany). Dilutions of 1:10^−3^, 1:10^−4^, and 1:10^−5^ were performed on each drain sample. Thus, the lower limit of detection was 10^3^ CFU/100 mL. *E. coli* concentrations for a given sample were calculated as the average of all quantifiable dilutions performed for that sample. All sample collection materials were sterilized with 96% alcohol and flamed to sterilize before and after use. All samples tested had an accompanying negative control.

### 2.4 Analysis

Chi-square (and Fisher's exact tests when expected cell frequencies were low) and one-way analysis of variance (ANOVA) tests were performed to assess differences in household sanitation as well as drain characteristics by neighborhood.

We hypothesized that drain characteristics (type of lining and composition, size) and precipitation (rainfall) would be important covariates to test in models estimating fecal contamination in drains (Berendes et al., 2018a; Chow et al., 2013; Ekklesia et al., 2015; Gretsch et al., 2015; Labite et al., 2010; Rochelle-Newall et al., 2015). Rainfall was characterized by: 1) the occurrence of rainfall during the 24 h before drain sample collection; and 2) concurrent rainfall during sample collection. All *E. coli* concentrations were log_10_ transformed and calculated per 100 mL. These factors were assessed in linear regression models adjusted for neighborhood.

Final models to assess associations between neighborhood and *E. coli* controlled for all covariates that: 1) indicated a meaningful association with drain *E. coli* concentration (p < 0.10) when adjusting for neighborhood; and 2) were not co-linear in hypothesized *a priori* directed acyclic graphs (Supplementary Information: Fig. S1). All models of *E. coli* concentrations, both unadjusted and adjusted, were generated using mixed-effects linear regression (lme4 package (Bates et al., 2014)) in R version 3.4.3 (R Foundation for Statistical Computing, Vienna, Austria (R Core Team, 2015)), using an alpha of 0.05 to assess statistical significance and a random effect for sampling location to account for locations where multiple samples were collected.

## 3 Results

### 3.1 Household demographics

Of the 150 households per neighborhood targeted for survey, along with the additional 50 targeted households in Chorkor (650 households in total), 29 households in Ringway Estates refused to participate, resulting in a total of 621 households with completed surveys across the four study neighborhoods in Accra, Ghana: Adabraka, Chorkor, Kokomlemle, and Ringway Estates.

Demographics and sanitation characteristics of surveyed households were compared across neighborhoods to assess heterogeneity (Table 1). Most households (78%) had a toilet. Household toilet coverage, as well as other sanitation characteristics including toilet type and number of households reporting sharing a toilet, varied significantly across neighborhoods. By observation, 77% of surveyed households had at least one contained toilet: mostly in Ringway (97%), Kokomlemle (92%), and Adabraka (88%). Only 48% of households had a contained toilet in Chorkor. Most contained toilets were flush toilets (93%). About one-third (37%) of surveyed households reported sharing their toilet with other households. Over half of all surveyed households (56%) reported using a public toilet at least once per month. Half of all households in Chorkor (52%) reported that they did not have a toilet, and most also reported using a public toilet at least once per month (74%).

**Table 1:** Household sanitation characteristics by neighborhood in Accra, Ghana.

	Adabraka^a^	Chorkor^b^	Kokomlemle	Ringway Estates^c^	Total^d^	p-value^e^
*Households surveyed*	150	200	150	121	621	
Average household size ±SD	14.5 ± 11.3	20.2 ± 16.3	12.6 ± 10.0	8.1 ± 6.7	14.6 ± 13.0	<0.001
No. Households with a toilet observed (%)	130 (89)	96 (48)	139 (93)	117 (97)	482 (78)	<0.001
No. HH's with at least 1 contained toilet (%)	128 (88)	95 (48)	138 (92)	117 (97)	478 (77)	<0.001
No. who report sharing toilet with ≥1 household/compound (%)	64 (43)	35 (18)	84 (56)	45 (37)	228 (37)	<0.001
Average No. of HH that share a single toilet ±SD	2.1 ± 3.7	1.7 ± 4.3	1.8 ± 2.4	1.1 ± 2.0	1.7 ± 3.2	0.090
No. (%) of Households using public toilets ≥1x/month	66 (44)	148 (74)	83 (55)	51 (42)	348 (56)	<0.001
Type of toilet/latrine in Household/Compound						<0.001
Flush toilet	120 (80)	77 (39)	131 (87)	116 (96)	444 (71)	
Pour flush	2 (1.3)	6 (3.0)	1 (0.7)	0	9 (1.4)	
Kumasi Ventilated-Improved Pit (KVIP)	4 (2.7)	6 (3.0)	2 (1.3)	0	12 (1.9)	
Ventilated Improved Pit (VIP)	2 (1.3)	5 (2.5)	4 (2.7)	0	11 (1.8)	
Traditional pit latrine	0	1 (0.5)	0	1 (0.8)	2 (0.3)	
Bucket/Pan	2 (1.3)	0	0	0	2 (0.3)	
No facility/bush/field	16 (11)	104 (52)	11 (7.3)	4 (3.3)	135 (22)	
Other	0	1 (0.5)	1 (0.7)	0	2 (0.3)	

a 4 households had toilets that were unable to be observed (refused).

b 1 household refused to respond to questions about public toilet use.

c 29 households refused the entire survey.

d Percentages may not add to exactly 100 due to rounding.

e Neighborhood-level differences were assessed for categorical variables (using Chi-square tests or Fisher's exact tests when expected cell frequencies < 5) and continuous variables (using one-way analysis of variance).

### 3.2 Environmental characteristics

Because of logistical constraints, 137/160 samples from 117/140 unique sampling sites were able to be collected and analyzed (Fig. 1): 27 sites (32 samples) in Adabraka, 35 sites (40 samples) in Chorkor, 28 sites (33 samples) in Kokomlemle, and 27 sites (32 samples) in Ringway Estates (Table 2). Drain characteristics, such as size and lining composition, differed significantly across neighborhoods. Sampled drains were uncovered and generally small (62% < 0.5 m wide at the sampled location), and most were lined with cement (88%). Water levels in drains were generally low: less than one-quarter filled when 129/137 drain samples were collected (Supplementary Information: Table S1). Rainfall during (2%) or the day before (17%) drain sample collection was rare (Table 2). Visible feces and animals were infrequently observed within 3 m of sampling location at time of sample collection (8% and 9%, respectively).

**Table 2:** Drain characteristics and drain sample collection in four study neighborhoods in Accra, Ghana.

	Adabraka	Chorkor	Kokomlemle	Ringway Estates	Total	p-value^a^
Unique drain sample sites (total samples)	27 (32)	35 (40)	28 (33)	27 (32)	117 (137)	
Average *E. coli* concentration in sampled drain locations (log_10_ CFU/100 mL) ± SD	4.7 ± 0.6	5.6 ± 0.9	4.9 ± 1.1	4.1 ± 0.8	4.9 ± 1.0	<0.001
95% confidence interval (CI)	4.5, 4.9	5.3, 5.9	4.5, 5.3	3.8, 4.4	4.7, 5.0	
Average drain size						<0.001
Small (<0.5 m wide) (%)	18 (67)	26 (74)	13 (46)	15 (56)	72 (62)	
Medium (0.5 – 1 m wide) (%)	7 (26)	5 (14)	15 (54)	9 (33)	36 (31)	
Large (>1 m wide) (%)	2 (7.4)	4 (11)	0	3 (11)	9 (7.7)	
Primary drain lining composition						0.024
Cement (%)	27 (100)	22 (63)	28 (100)	26 (96)	103 (88)	
Stones (%)	0	2 (5.7)	0	0	2 (1.7)	
Dirt (%)	0	6 (17)	0	0	6 (5.1)	
Mixed (%)	0	5 (14)	0	1 (3.7)	6 (5.1)	
Rained during sample collection	2 (7.4)	0	0	0	2 (1.7)	0.103
Rained the day before sample collection	20 (74)	0	0	0	20 (17)	<0.001
Feces observed within 3 m of sample location	1 (3.7)	5 (14)	3 (11)	0	9 (7.7)	0.141
Animals observed within 3 m of sample location	2 (7.4)	7 (20)	1 (3.6)	0	10 (8.5)	0.028

a Neighborhood-level differences assessed for categorical variables (using chi-square tests, or Fisher's exact tests when expected cell frequencies < 5) and continuous variables (using one-way analysis of variance).

**Fig. 2:**
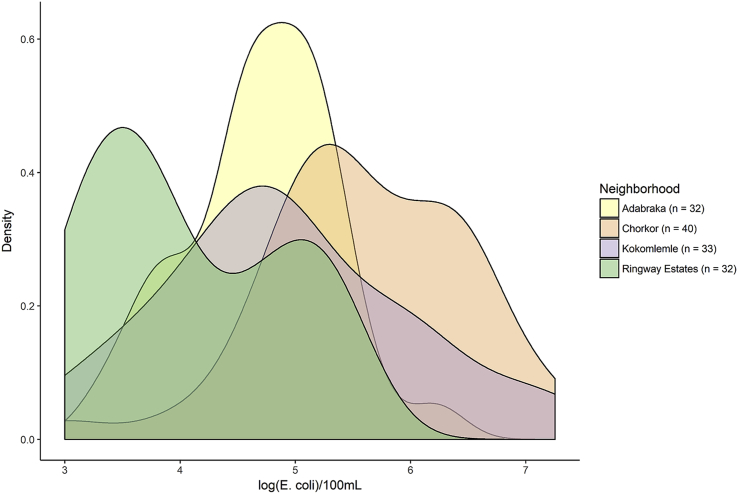
Neighborhood-level distributions of *E. coli* in drains.

All drain samples had *E. coli* concentrations quantifiable above the lower limit of detection. None of the negative controls run tested positive for *E. coli.* The geometric mean of *E. coli* concentrations in drain samples was 4.9 log_10_ CFU/100 mL (standard deviation (SD): 1.0 log_10_ CFU/100 mL, Table 2). Drains in Chorkor had the highest geometric mean concentrations of *E. coli* (5.6 log_10_ CFU/100 mL), while drains in Ringway Estates had the lowest (4.1 log_10_ CFU/100 mL). Geometric means of *E. coli* concentrations in drains in Adabraka and Kokomlemle were similar (4.7 log_10_ CFU/100 mL vs. 4.9 log_10_ CFU/100 mL). In addition to between-neighborhood differences in *E. coli* concentrations, within-neighborhood *E. coli* concentrations varied widely, generally with ranges spanning >2 log_10_ CFU/100 mL (Fig. 2). Among the subset of sampling locations (n = 5 per neighborhood) where multiple samples were collected within the same day, differences in *E. coli* concentrations were 0.7 log_10_ CFU/100 mL on average (95% confidence interval: 0.4, 1.0; Supplementary Information: Table S2).

### 3.3 Variation in *E. coli* concentration by neighborhood, drain infrastructure, and rainfall

Linear regression was used to examine associations between drain infrastructure, rainfall conditions, and environmental conditions at drain sample sites, and fecal contamination (*E. coli* concentration) in drains, adjusting for neighborhood (Table 3a). *E. coli* concentrations in small drains (<0.5 m wide) were 0.8 log_10_ CFU/100 mL lower than those in the largest drains (>1 m wide, p < 0.01). Assessed at an aggregate level using linear regression, samples collected in the morning did not vary significantly from those collected in the afternoon.

**Table 3:** Differences in *E. coli* concentrations (in log_10_ CFU/100 mL) of sample drain sites by drain infrastructure, seasonality, environmental conditions, and neighborhood.

		Mixed effects linear regression model (n = 137)		
a) Risk factor estimate^a^	β^b^	SE	95% CI^c^	p-value
Drain size				
Small (<0.5 m wide)	−0.80	0.27	−1.33, −0.28	0.004
Medium (0.5–1.0 m wide)	−0.11	0.30	−0.67, 0.46	0.712
Large (>1.0 m wide)	Ref.	Ref.	-	-

Drain lining				
Dirt	Ref.	Ref.	-	-
Cement	−0.65	0.39	−1.40, 0.09	0.098
Stones	0.59	0.70	−0.75, 1.94	0.399
Mixed	−0.29	0.48	−1.23, 0.63	0.545

Rain during collection	−0.09	0.64	−1.32, 1.15	0.891

Rain last day	0.28	0.34	−0.38, 0.93	0.417

Feces near	0.49	0.30	−0.10, 1.09	0.112

Animals near	0.05	0.26	−0.45, 0.57	0.854

Sampled in morning (v. afternoon)	−0.06	0.15	−0.35, 0.25	0.683
b) Adjusted^d^ neighborhood estimate	β^b^	SE	95% CI^c^	p-value
Ringway Estates	Ref.	Ref.	-	-
Adabraka	0.79	0.20	0.40, 1.18	<0.001
Kokomlemle	0.84	0.20	0.44, 1.24	<0.001
Chorkor	1.59	0.21	1.16, 2.01	<0.001
*P-values for pairwise post-hoc assessments of neighborhood-neighborhood differences*				
Ringway – Adabraka				0.001
Ringway – Kokomlemle				<0.001
Ringway - Chorkor				<0.001
Adabraka – Kokomlemle				0.994
Adabraka – Chorkor				0.001
Kokomlemle – Chorkor				0.004

a Adjusted for neighborhood.

b log_10_ CFU/100 mL.

c 95% confidence interval for the estimate (beta).

d Adjusted for drain lining, drain size, and including a random effect for time of collection (morning or afternoon for sites with two samples collected during the day).

Neighborhood-level *E. coli* concentrations in drains were compared in models that adjusted for drain lining and drain size (Table 3b). Compared with *E. coli* concentrations in drains in Ringway Estates, concentrations in Adabraka, Kokomlemle, and Chorkor drains were significantly higher. Means and 95% confidence intervals (95% CI) for log_10_ CFU/100 mL E*. coli* concentrations in adjusted models, by neighborhood, were 4.6 in Ringway Estates (95% CI: 4.1–5.0), 5.4 in both Adabraka and Kokomlemle (95% CI: 4.9–5.9 for each), and 6.2 in Chorkor (95% CI: 5.8–6.5). Using pairwise post-hoc tests with a Tukey adjustment, differences in *E. coli* concentrations in drains between each pair of neighborhoods were highly significant (p < 0.01), with the exception of Adabraka and Kokomlemle.

## 4 Discussion

Overall, we observed that neighborhoods with high (85–97%) coverage of contained household sanitation still had high average *E. coli* concentrations in their drains: ≥4 log_10_ CFU/100 mL. These results suggest that there is a limited extent to which isolated (single neighborhood) high coverage of household sanitation facilities classified as “contained” (designed to have safe containment of feces in toilets that prevent direct discharge into open drains) can reduce fecal contamination in urban environments. Further, we observed mean *E. coli* concentrations in drains varied by about 1.5 log_10_ CFU/100 mL across neighborhoods and were generally: a) lower in the high (>85%) sanitation coverage neighborhoods (with high-income and lower population density); and b) higher in the low (<50%) sanitation coverage neighborhood (with low-income, higher population density). However, other factors (e.g. animal contributions to fecal contamination; availability of safe emptying, transport, and treatment of onsite waste; sanitation coverage and management in nearby neighborhoods, population density) may also contribute to open drain contamination. Thus, household sanitation coverage alone may not be sufficient to reduce open drain contamination in low-income, urban settings.

This study builds on previous studies of environmental fecal contamination in dense, generally poor, urban neighborhoods in low- and middle-income countries (LMICs) (D. Berendes et al., 2017; Berendes et al., 2018a; Exum et al., 2016); however, it is unique in its comparison of environmental conditions in low-income, low-coverage neighborhoods to a high-income, high-coverage neighborhood in the same city. Concentrations of *E. coli* observed in open drains in this study are consistent with those from low-income, informal, urban settlements in Kampala, Uganda in 2013 (Katukiza et al., 2013), but lower than those from previous assessments of open drains in Accra, Ghana (Berendes et al., 2018a; Labite et al., 2010).

Despite lower *E. coli* concentrations in neighborhoods with high (sometimes >90%) sanitation coverage, open drains in all neighborhoods—even those with almost 100% coverage of sanitation facilities at households—had concentrations high enough to constitute a public health hazard, especially given the prevalence of open drainage. Mean *E. coli* levels of 4.1–5.6 log_10_ CFU/100 mL are lower than in drains of uniformly low-income neighborhoods of Accra (8.5 log_10_ CFU/100 mL) (Berendes et al., 2018a), but these drains may still carry large pathogen loads from human and animal sources (Blom, 2015). For context, the lowest concentrations of *E. coli* observed in open drains in this study were still 10–100 fold greater than USEPA regulations for wastewater discharge into recreational water bodies that humans contact (EPA, 2012). Volumes of water in drain are smaller than those in recreational water thereby allowing for minimal dilution prior to human contact. Given the ubiquitous, interconnected nature of drains, fecal contamination in one neighborhood may be the result of inflow from surrounding neighborhoods, thus well-coordinated sanitation interventions to contain feces at the city-level are imperative (Eisenberg et al., 2012; Peal et al., 2014a, 2014b). Preventing contact with open drains—whether due to environmental factors (e.g. flooding) or personal behavior (e.g. accidental contact while a child is playing)—is also important to protect public health (Berendes et al., 2019; Gretsch et al., 2015; Katukiza et al., 2013; Labite et al., 2010).

We observed evidence that increasing coverage of onsite sanitation may be associated with lower *E. coli* concentrations in open drains. Although both humans and animals may contribute *E. coli* to drains, we observed significant variation in *E. coli* concentrations by neighborhood (purposively chosen for variation in sanitation coverage) and did not observe variation in *E. coli* concentrations with the presence or absence of animals near drains, although the small number of drains with animals observed nearby (10) limits this inference. High coverage of contained sanitation—if in use, properly functioning, and regularly emptied, and even if shared—should sequester human feces from the environment, as first described in the classic ‘F diagram’ of WASH-associated fecal exposure pathways (Wagner and Lanoix, 1958). In contrast, households without contained sanitation may be discharging feces into the environment at the household (e.g. poorly cited toilet, or absence of a toilet/open defecation directly into drains) and downstream. Previous evidence, limited to low-income neighborhoods of Accra with high *E. coli* concentrations in drains, also suggests that within-neighborhood clusters with high coverage of contained sanitation may have lower relative levels of fecal contamination in drains (Berendes et al., 2018a). We note, however, that the presence of a toilet alone does not ensure its use and we did not map open defecation except to visually assess whether feces was present within 3 m of the sampling site. We also were unable to characterize whether the household sanitation meeting observation-based criteria for ‘contained’ sanitation had sufficient onsite containment vs. offsite overflow. Also, the final fate of the fecal waste from survey households was not documented, thus we could not evaluate the safe management of feces along the entire sanitation chain. A significant portion of contained, improved, or other systems meeting such criteria may still end up discharging unsafely or otherwise as untreated fecal waste (Baum et al., 2013).

Our observation that *E. coli* concentrations varied significantly, by about 0.8 log_10_ CFU/100 mL, between the highest sanitation coverage neighborhood (Ringway Estates, 97%) and other high sanitation coverage neighborhoods (Adabraka and Kokomlemle, 85–93%) also suggests that factors other than the type and coverage of household toilets may affect *E. coli* concentrations in drains in these neighborhoods. These factors may include the geographic location of the neighborhood, as well as household income and population density. For example, given that drains are geographically interconnected across the city, drains in Adabraka or Kokomlemle near other neighborhoods with lower sanitation coverage likely received drain water with more fecal contamination than the drain water received by Ringway Estates (CHF International et al., 2010; Weeks et al., 2012), though we did not explicitly map between-neighborhood drainage flow. Income variation across neighborhoods, correlated with sanitation coverage (Nelson and Murray, 2008; Weeks et al., 2012), may have affected fecal contamination in multiple ways. Among them, residents of Ringway Estates may have been better able to pay the high user-borne costs associated with regular emptying and maintenance of onsite sanitation (Berendes and Brown, 2018; Dodane et al., 2012), had fewer animals, and/or were better able to manage animals and their large onsite fecal hazards (Berendes et al., 2018b). Though no questions about animal or human feces management were included in household surveys, no sampling sites in Ringway Estates had visible feces or animals observed within 3 m of the sampling location, in contrast to Adabraka and Kokomlemle.

Finally, population density—like income—is difficult to separate from sanitation coverage in these neighborhoods. Kokomlemle and Adabraka were the only neighborhoods in the same category of sanitation coverage and income, but different categories of population density (CHF International et al., 2010; Ghana Statistical Service, 2014); however, they did not differ significantly in *E. coli* concentrations in drains. Despite our findings, population density may still affect not only disease transmission, but also access to and use of safe disposal of human feces, especially in urban settings (Isunju et al., 2011).

Taken together, these results imply that reducing fecal contamination in neighborhood environments, such as open drains, requires multi-scale and multi-faceted interventions. If—at a city level—less than 10% of fecal sludge is estimated to be safely managed and open drains with fecal waste are ubiquitous (Mansour and Esseku, 2017), the impact of neighborhood-level interventions to contain fecal waste will be limited without broader, concurrent initiatives. Additionally, these results are consistent with other evidence of associations between income or socioeconomic status and urban environmental quality in Accra (Fobil et al., 2010), underscoring the interdisciplinary nature of fecal contamination in the urban environment.

This study has several strengths and limitations that should be considered. While sampling drains spatially at random is a study strength, we did not have the statistical power to measure sub-neighborhood variation in *E. coli* concentrations in drains, including with variation in specific types of sanitation systems. This limits our ability to provide more direct evidence of sanitation coverage as the driver of variation in *E. coli* concentrations in drains, though our previous sub-neighborhood evidence within low-income neighborhoods in Accra supports this finding (Berendes et al., 2018a). Despite the timing of sample collection within the calendar year, there was almost no rainfall during the 2016 rainy season when samples were collected, which suggests most of the sampled drain water was coming from sanitation systems and greywater. Sampling during periods of rainfall may have provided a more accurate estimate of contamination levels associated with flooding or other runoff. Most of the samples in this study were collected in the morning; however, collection of samples across the entire day may have provided a better approximation of diurnal or other temporal variations in concentration (Ekklesia et al., 2015). Use of *E. coli* as an indicator of fecal contamination was not accompanied by microbial source tracking data to identify animal fecal contamination, which would have provided important evidence for sources of drain contamination.

## 5 Conclusions

This analysis suggests that fecal contamination in drains varies significantly, by about 1.5 log_10_ CFU/100 mL, across neighborhoods of Accra, Ghana with varying sanitation (by almost 50 percentage points), income, and population density., These results suggest that high coverage of sanitation facilities that safely contain waste onsite at the neighborhood-level, alongside higher income and population density, may only reduce levels of fecal contamination in the surrounding environment to a limited extent. We observed that even high-income, low population density neighborhoods with almost complete (97%) coverage of household-level sanitation facilities had >4 log_10_ CFU/100 mL E*. coli* in open drains, sufficient to present a public health hazard. Although variation in fecal contamination may be influenced by neighborhood sanitation conditions, other factors may contribute. It is important to recognize that other scales of interventions, including improvements in sanitation and fecal sludge management at the city-level, may be necessary to address additional drivers of environmental and drain contamination. These findings underscore the interconnectedness of excreta management from multiple sources in urban neighborhoods and the importance of citywide inclusive sanitation to reduce fecal contamination in urban environments.

## Author contributions

Conceptualization, DMB, AEK, HY, SR, KR, and CLM.; Methodology, DMB, AEK, HY, LA, SR, KR, BD, JA, and CLM; Software, DMB, JM, SR, YW.; Validation, DMB, LdM, AEK, and HY; Formal Analysis, DMB, LdM, HY, LA, YW, BD; Investigation, LdM, HY, LA, BD.; Resources, SR, KR, JA, and CLM; Data Curation, LdM, HY, JM, YW; Writing – Original Draft Preparation, DMB, LdM, AEK; Writing – Review & Editing, all authors; Visualization, DMB and LdM; Supervision, AEK, JA, and CLM; Project Administration, HY, SR, and YW; Funding Acquisition, DMB, HY, SR, KR, and CLM.

## Funding

The study was financially supported by the Bill & Melinda Gates Foundation, grant no. OPP1016151.

## Disclaimer

The findings and conclusions of this report are those of the authors and do not necessarily represent the official position of the Centers for Disease Control and Prevention.

## Declaration of competing interest

The authors declare no conflicts of interest. The funders had no role in the design of the study; in the collection, analyses, or interpretation of data; in the writing of the manuscript, or in the decision to publish the results.
