# Supplementary material for: Variation in E. coli concentrations in open drains across neighborhoods in Accra, Ghana: The influence of onsite sanitation coverage and interconnectedness of urban environments
Source: Int J Hyg Environ Health. 2020 Mar;224:113433. doi: 10.1016/j.ijheh.2019.113433 (PMC6996153; doi:10.1016/j.ijheh.2019.113433)
Supplement: Multimedia component 2 [file mmc2.docx]

Supplementary Information

*Figure S1: Hypothesized causal relationship between sanitation indicator variables and fecal contamination in open drains via directed acyclic graph (DAG)*


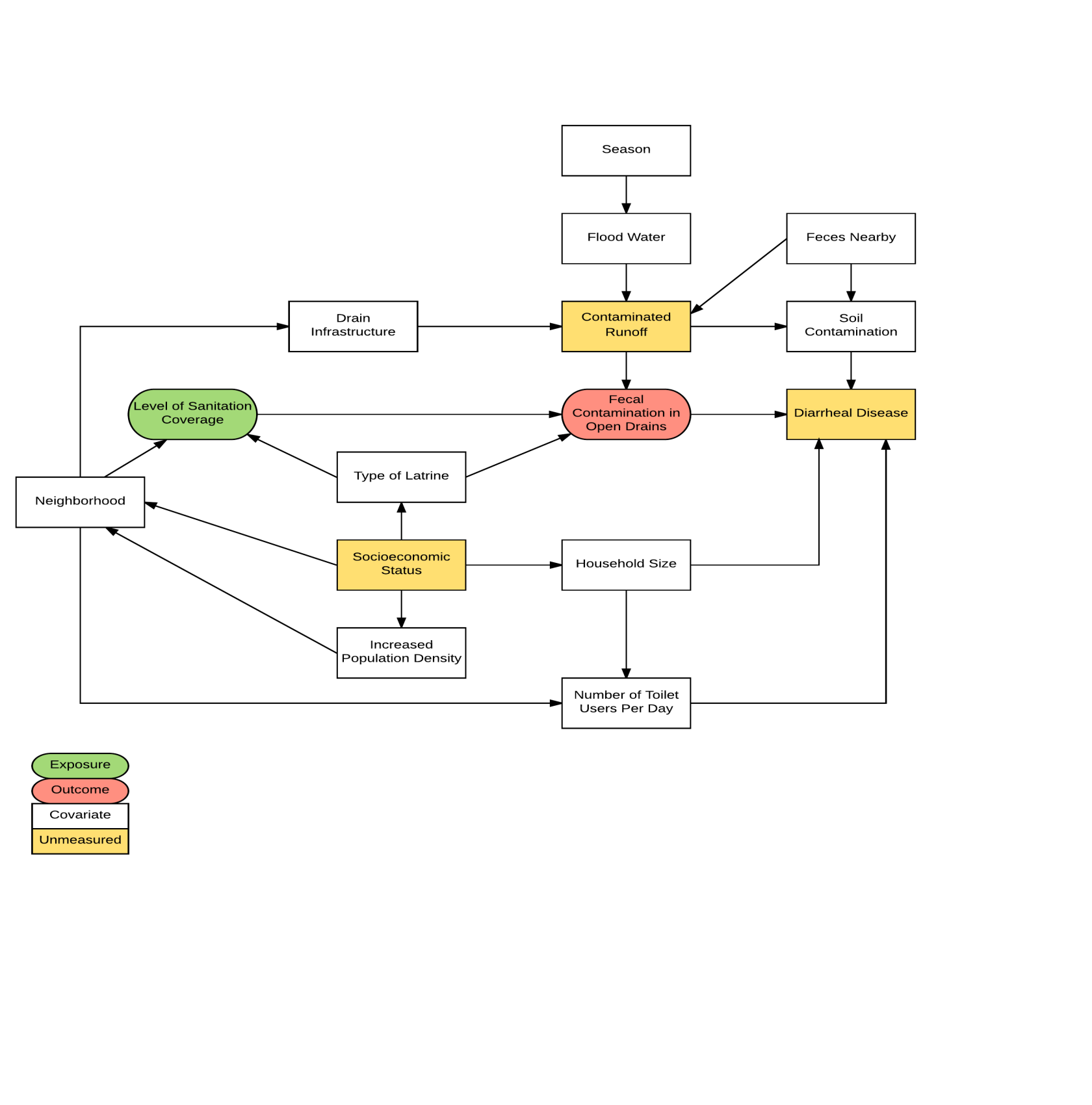


| **Table 1. Water levels in drain sampling locations by neighborhood, Accra, Ghana^1.^** | | | | | |
| --- | --- | --- | --- | --- | --- |
|  | **Adabraka** | **Chorkor** | **Kokomlemle** | **Ringway** | **Total** |
| Average drain size |  |  |  |  |  |
| Small (<0.5m wide) (%) | 23 (72) | 31 (78) | 16 (48) | 16 (50) | 86 (63) |
| Nearly dry | 0 | 1 | 0 | 0 | 1 |
| Water level < ¼ full | 23 | 29 | 16 | 16 | 84 |
| Water level ~ ½ full | 0 | 1 | 0 | 0 | 1 |
| Water level > ¾ full | 0 | 0 | 0 | 0 | 0 |
| Medium (0.5 – 1m wide) (%) | 7 (22) | 5 (13) | 17 (52) | 12 (38) | 41 (30) |
| Nearly dry | 0 | 1 | 0 | 0 | 1 |
| Water level < ¼ full | 7 | 4 | 16 | 10 | 37 |
| Water level ~ ½ full | 0 | 0 | 1 | 2 | 3 |
| Water level > ¾ full | 0 | 0 | 0 | 0 | 0 |
| Large (>1m wide) (%) | 2 (6.3) | 4 (10) | 0 | 4 (13) | 10 (7) |
| Nearly dry | 0 | 0 | 0 | 0 | 0 |
| Water level < ¼ full | 1 | 4 | 0 | 1 | 6 |
| Water level ~ ½ full | 1 | 0 | 0 | 3 | 4 |
| Water level > ¾ full | 0 | 0 | 0 | 0 | 0 |
| ^1^Percentages may not add to 100%, by column, due to rounding. | | | | | |

| **Table 2. Within-day differences in *E. coli* concentrations in drains among a subset of drain sampling locations (n = 5 per neighborhood) by neighborhood, Accra, Ghana^.^** | | | | | |
| --- | --- | --- | --- | --- | --- |
|  | **Adabraka** | **Chorkor** | **Kokomlemle** | **Ringway** | **Total** |
| Geometric mean difference | 0.7 | 0.3 | 0.9 | 1.0 | 0.7 |
| Standard deviation | 1.0 | 0.3 | 0.8 | 0.6 | 0.7 |
| 95% Confidence interval**^1^** | -0.5, 2.0 | -0.2, 0.7 | -0.1, 1.8 | 0.2, 1.7 | 0.4, 1.0 |
| **^1^**Confidence intervals may cross 0 despite taking the absolute value of the difference (smaller value subtracted from larger value). | | | | | |
